# High resolution detectors for whole-body PET scanners by using dual-ended readout

**DOI:** 10.1186/s40658-022-00460-4

**Published:** 2022-04-21

**Authors:** Zheng Liu, Ming Niu, Zhonghua Kuang, Ning Ren, San Wu, Longhan Cong, Xiaohui Wang, Ziru Sang, Crispin Williams, Yongfeng Yang

**Affiliations:** 1 Paul C. Lauterbur Research Center for Biomedical Imaging, Shenzhen Institutes of Advanced Technology, Chinese Academy of Sciences, Shenzhen, 518055 China; 2 European Centre for Nuclear Research (CERN), Geneva, Switzerland

**Keywords:** PET detector, Depth of interaction, Time of flight, High resolution, Silicon photomultiplier

## Abstract

**Background:**

Most current whole-body positron emission tomography (PET) scanners use detectors with high timing resolution to measure the time-of-flight of two 511 keV photons, improving the signal-to-noise ratio of PET images. However, almost all current whole-body PET scanners use detectors without depth-encoding capability; therefore, their spatial resolution can be affected by the parallax effect.

**Methods:**

In this work, four depth-encoding detectors consisting of LYSO arrays with crystals of 2.98 × 2.98 × 20 mm^3^, 2.98 × 2.98 × 30 mm^3^, 1.95 × 1.95 × 20 mm^3^, and 1.95 × 1.95 × 30 mm^3^, respectively, were read at both ends, with 6 × 6 mm^2^ silicon photomultiplier (SiPM) pixels in a 4 × 4 array being used. The timing signals of the detectors were processed individually using an ultrafast NINO application-specific integrated circuit (ASIC) to obtain good timing resolution. The 16 energy signals of the SiPM array were read using a row and column summing circuit to obtain four position-encoding energy signals.

**Results:**

The four PET detectors provided good flood histograms in which all crystals could be clearly resolved, the crystal energy resolutions measured being 10.2, 12.1, 11.4 and 11.7% full width at half maximum (FWHM), at an average crystal depth of interaction (DOI) resolution of 3.5, 3.9, 2.7, and 3.0 mm, respectively. The depth dependence of the timing of each SiPM was measured and corrected, the timing of the two SiPMs being used as the timing of the dual-ended readout detector. The four detectors provided coincidence time resolutions of 180, 214, 239, and 263 ps, respectively.

**Conclusions:**

The timing resolution of the dual-ended readout PET detector was approximately 20% better than that of the single-ended readout detector using the same LYSO array, SiPM array, and readout electronics. The detectors developed in this work used long crystals with small cross-sections and provided good flood histograms, DOI, energy, and timing resolutions, suggesting that they could be used to develop whole-body PET scanners with high sensitivity, uniform high spatial resolution, and high timing resolution.

## Background

Since the revival of the time-of-flight (TOF) positron emission tomography (PET) scanner in the early 2000s, it has been offered by most medical device vendors and has become a routine nuclear medicine diagnosis tool [1–4]. TOF information improves the signal-to-noise ratio (SNR) of the reconstructed PET images, thus improving diagnostic accuracy. Owing to their compactness, high photon detection efficiency, good timing properties, low operating voltage, and low cost, silicon photomultipliers (SiPMs) [5–8] are currently the first choice photodetector for commercial clinical TOF-PET scanners [9–13]. The timing resolution of TOF-PET scanners has continued to improve over the last 15 years, from 500–600 ps for the first-generation TOF-PET scanners [14, 15] to 200–400 ps for current state-of-the-art TOF-PET scanners [9, 12], partly due to the use of SiPM photodetectors and their improved timing properties. Meanwhile, improving the timing resolution of PET detectors has been a hot research topic for the past 15 years [4, 16, 17]. New detector designs [18, 19], electronic techniques [20], photon generation mechanisms [21–23], and photodetectors [24] have been studied to improve the timing resolution of PET detectors. To date, the best timing resolution of ~ 30 ps full width at half maximum (FWHM) has been obtained using detectors consisting of a Cerenkov radiator (PbF_2_ glass) and a microchannel plate [25].

The uncertainty of the depth of interaction (DOI) measurement degrades the spatial resolution of a PET scanner and causes nonuniformity of the spatial resolution within its field of view. Moreover, because the DOI effect is larger for PET scanners with smaller detector ring diameters and higher position resolution detectors, the development of depth-encoding PET detectors over the past years has been focused primarily on the high position resolution detectors required for preclinical, dedicated breast and brain PET scanners. Various DOI-encoding PET detector techniques [26, 27]—such as multilayer crystal arrays [28–30], dual-ended readout of pixelate crystal arrays [31, 32], monolithic scintillators [11, 33] and single-ended readout of crystal arrays measuring light sharing between crystals [34–36]—have been developed, some of which have been successfully used in PET scanners [30, 37–40].

Although the DOI effect of whole-body PET scanners is smaller than that of small-animal and dedicated-brain PET scanners, it still degrades the spatial resolution of whole-body PET scanners at positions with large radial offsets. For example, the radial spatial resolutions at radial offsets of 1, 10, and 20 cm are 3.5, 4.5, and 5.8 mm FWHM for the Siemens Biograph Vision PET scanner [12] and 4.4, 5.8, and 8.4 mm FWHM for the GE SIGNA PET/MRI scanner [41] if filtered back-projection reconstruction is used. The DOI effect increases as the axial length increases and the crystal cross-section decreases; these are the current trends in whole-body PET scanner developments [42–44]. PET detector techniques with good DOI encoding capability and timing resolution have been investigated recently for whole-body PET scanners [45–48]. The dual-ended readout of a pixelated scintillator array is a well-studied depth-encoding technique that can resolve very small crystals and provide good DOI resolution [49, 50]. Several previous studies have also developed dual-ended readout PET detectors to simultaneously achieve precise DOI and timing resolution using single crystal and small crystal arrays [51–54].

In this work, four PET detectors consisting of LYSO arrays of different crystal cross-sections and lengths that are dual-ended read using SiPM arrays with a 6 × 6 mm^2^ pixel area were evaluated. Flood histograms, the energy resolution, DOI resolution, and coincidence time resolution (CTR) of the detectors are presented. SiPMs with a pixel size of 6 mm were used in this work because PET detectors using such SiPMs require a lower number of electronic channels. It has also been shown that crystals with a cross-section as small as 1.2 mm could be resolved using a SiPM array consisting of 6 × 6 mm^2^ SiPMs [55].

## Methods

### Detector design

**Fig. 1:**
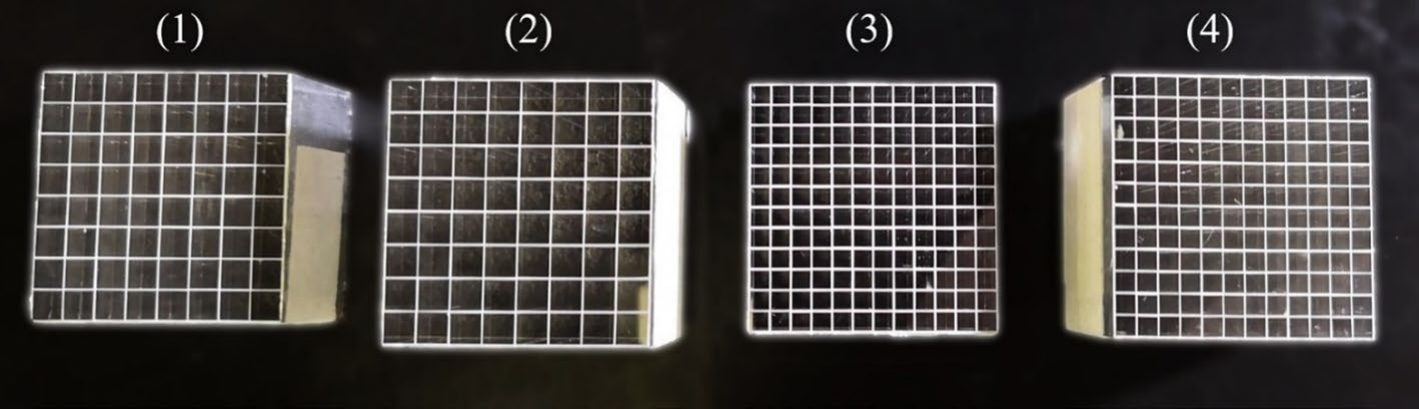
Photo of the four LYSO crystal arrays evaluated in this work, the crystal sizes of the four arrays being (1) 2.98 × 2.98 × 20 mm^3^, (2) 2.98 × 2.98 × 30 mm^3^, (3) 1.95 × 1.95 × 20 mm^3^, and (4) 1.95 × 1.95 × 30 mm^3^

Four LYSO crystal arrays manufactured by EBO Crystal Inc. (Shanghai, China) were evaluated in this study; a photograph of the four arrays is shown in Fig. 1. All surfaces of the LYSO crystals were polished, and the crystals were separated by 0.1-mm-thick barium sulphate (BaSO4) reflectors. The outside of the crystal arrays were wrapped with aluminum foil to hold the crystals together. Detailed information on the four crystal arrays is provided in Table 1.

**Table 1:** Detailed information of the four LYSO arrays

Detector #	Crystal array	Crystal size (mm^3^)	
1	8 × 8	2.98 × 2.98 × 20	
2	8 × 8	2.98 × 2.98 × 30	
3	12 × 12	1.95 × 1.95 × 20	
4	12 × 12	1.95 × 1.95 × 30	

LYSO arrays 1 and 2 were 8 × 8 arrays with a crystal cross-section of 2.98 × 2.98 mm^2^ and crystal lengths of 20 and 30 mm, respectively. LYSO arrays 3 and 4 were 12 × 12 arrays with a crystal cross-section of 1.95 × 1.95 mm^2^ and crystal lengths of 20 and 30 mm, respectively. Each crystal array had an end area of 24.6 mm^2^ that matched the active area of the SiPM array. The SiPM arrays were coupled to the crystal arrays either directly or using thin light guides (used between the crystal arrays and SiPM arrays to improve the identification of the edge crystals by increasing the scintillation photon spread). The light guides were made of K9 glass with and without grooves and had a thickness of 1 mm, area of 25.2 × 25.2 mm^2^ and refractive index of 1.5. Xiameter PMX-200 silicon oil (Dow Corning Corp., USA) was used between the scintillator and SiPM arrays, the scintillator array and light guide, and the light guide and SiPM array.

**Fig. 2:**
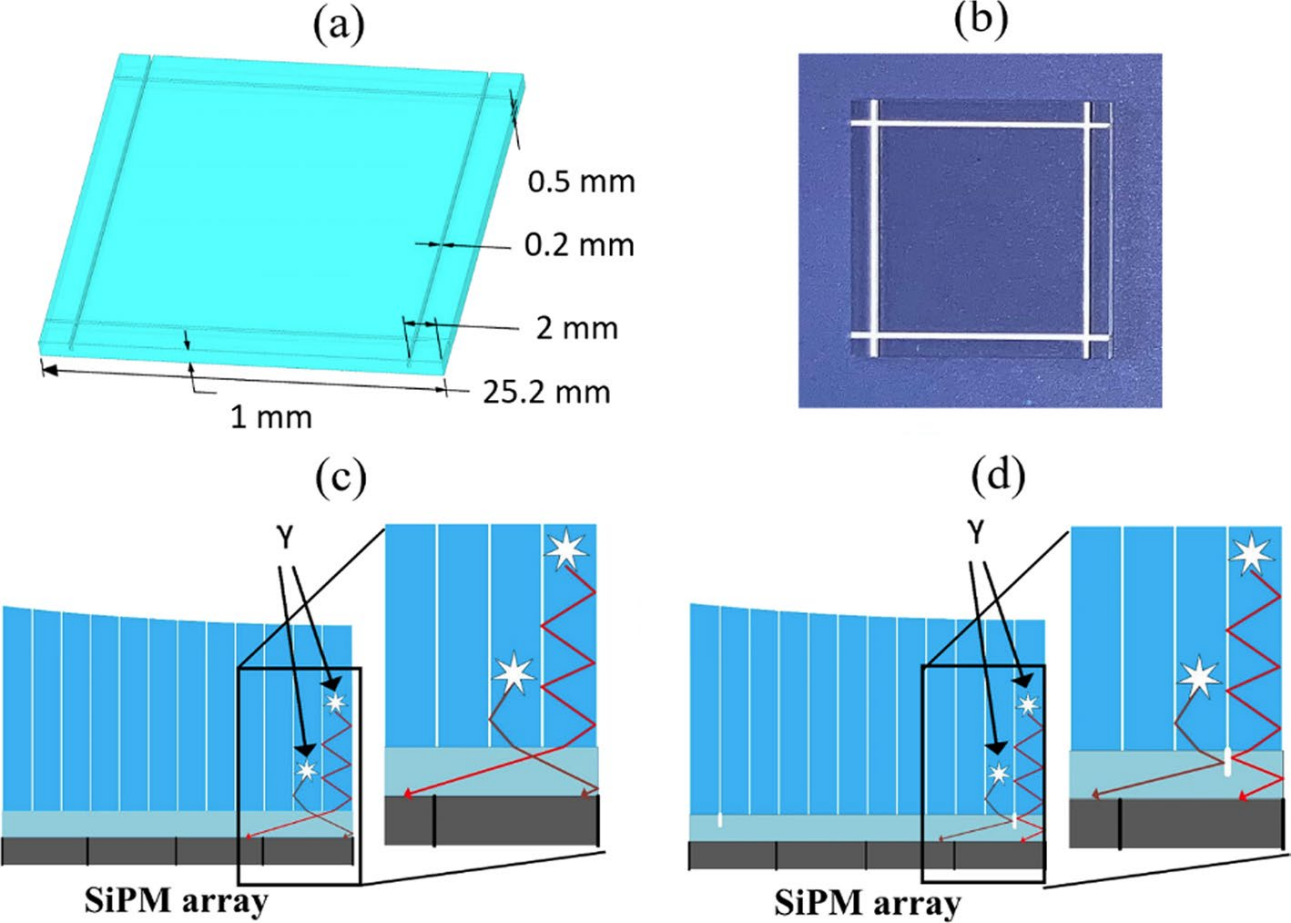
**a** Mechanical design, **b** photo of the light guide with grooves, and **c**–**d** illustration of how photon transport in the edge crystals and light guide improves the identification of the edge crystals

Figure 2 shows the design and a top-view photo of the light guide with grooves, and an illustration of how photon transport in the edge crystals and the light guide improves the identification of edge crystals. At 2 mm from the edges, four grooves of 0.2 mm wide and 0.5 mm deep are raw cut into the light guide and filled with BaSO4 reflector [56, 57]. Using the grooves in the light guide, more scintillation photons produced by interactions occurring in the second row (or column) of the crystals are detected by the second row (or column) of the SiPMs, and more scintillation photons produced by interactions occurring in the first row (or column) of the crystals are detected by the first row (or column) of the SiPMs. The result is that the identification of the edge of two rows (or columns) of the crystals improves.

**Fig. 3:**
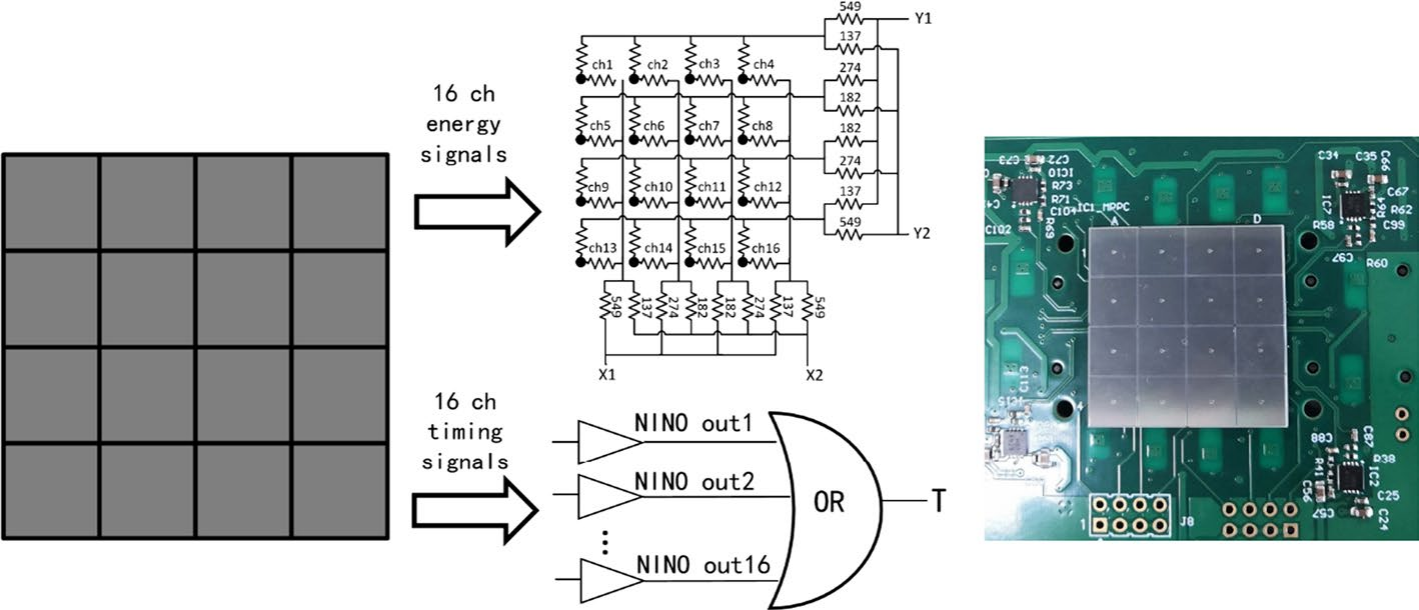
(left) The schematic of the signal readout of a 4 × 4 SiPM array, (right) photo of the SiPM array with the readout circuit board

A 4 × 4 SiPM array (Hamamatsu S14160-6050HS-04) with a 6 × 6 mm^2^ pixel size and 50 × 50 μm^2^ SPAD size was used to read out the LYSO arrays—the SiPM array having a total area of 25.0 × 25.0 mm^2^ and an active area of 24.6 × 24.6 mm^2^. To maintain good timing, the timing and energy signals from the SiPM arrays were processed separately. The timing signals from each SiPM were read individually and processed using a NINO application-specific integrated circuit (ASIC) [58, 59], as shown in Fig. 3.

For each SiPM array, the 16 logic timing output signals from the NINO ASIC were connected via an OR gate with a multiplexing ratio of 16:1—that is, the timing signal of the SiPM array was the earliest timing signal of the 16 SiPM pixels. The logic timing signals of the two SiPM arrays—which have a rise time of ~ 2 ns from baseline to the maximum amplitude (− 0.8 V)—were sent to a 2.5 GHz digital oscilloscope (DPO7254C, Tektronix Inc., OR, USA) with a sampling rate of 10 GS/s. The position at which the timing signal rises to half-height was then obtained by linear interpolation, generating the time stamp of that signal. The NINO ASIC allows for an 8-channel input signal charge measurement using a time-over-threshold technique with excellent timing resolution at a very high rate, while simultaneously providing very low noise performance and power consumption characteristics per channel. In a previous study [58], a root-mean-square timing jitter of 20 ps was measured for the NINO channel. The contribution of the NINO ASIC-based electronics to the CTR of the detectors was also measured in this work by sending 0.5 V square wave pulses with a 22 ps FWHM channel-to-channel jitter to two electronic channels using a Keysight 81150A pulse generator (Keysight Technologies, CA, USA), an FWHM timing resolution of 48 ps being obtained.

The energy signals of the 16 pixels of a SiPM array were read using a row and column summing circuit to form four position-encoding energy signals (X_1_, X_2_, Y_1_, Y_2_) that provide both position and energy information [60]. The four energy signals were amplified using an AD8045 amplifier (Analog Devices Inc., MA, USA) and digitized using an 8-channel data acquisition (DAQ) board (PD2-MFS-8 2M/14, United Electronic Industries Inc., MA, USA), the maximum total sampling rate of the board being 2 MS/s. The board was connected to a PC via a peripheral component interconnect bus. The software package for the DAQ system was developed at UC Davis and was described in detail in [61]. The oscilloscope and DAQ board were synchronized using the same event trigger signal. The SiPMs read out each side of the crystal array; thus, the SiPM signals always occurred together. An event trigger was generated by the oscilloscope from the SiPM signals; thus, the oscilloscope SNCY-OUT could be used to trigger the 8-channel DAQ board. In this manner, the energy and timing information were synchronized and correlated.

### Experimental setups and measurements

**Fig. 4:**
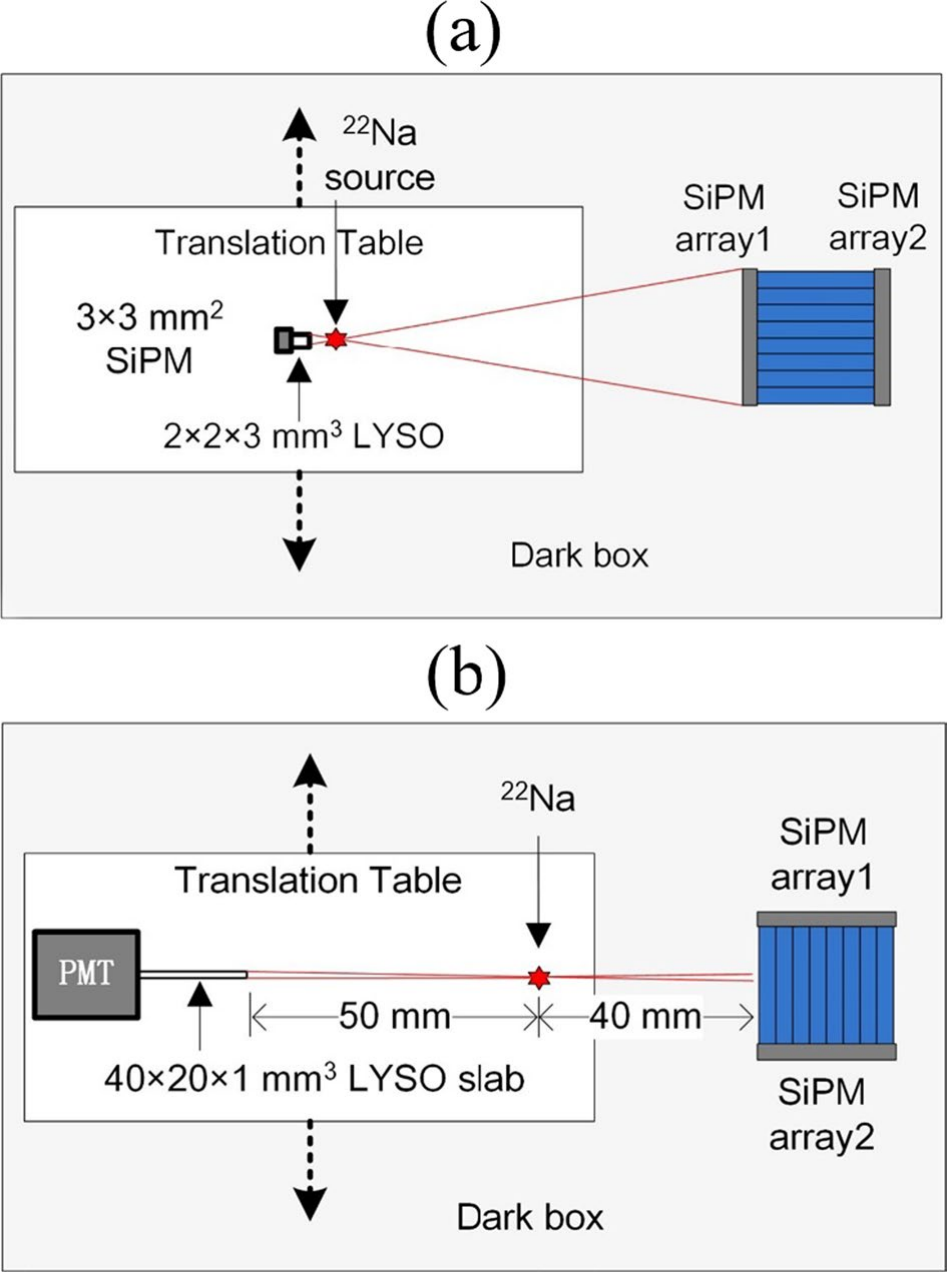
**a** Experimental setup for the flood histogram, energy resolution, and timing resolution measurements, the reference detector consisting of a 2 × 2 × 3 mm^3^ LYSO crystal read out using a 3 × 3 mm^2^ SiPM, **b** experimental setup for the DOI resolution measurements, the reference detector consisting of a 40 × 20 × 1 mm^3^ LYSO slab read using a single-channel PMT

The four PET detectors were measured using two different experimental setups. Figure 4a shows the experimental setup for the flood histogram, energy resolution, and timing resolution measurements. A ^22^Na point source of 0.25 mm diameter and an activity of 9.53 μCi was placed between the test PET detector and the reference detector. The distance from the point source to the front of the test PET detector was 70 mm, the reference detector being placed ~ 4 mm from the source to obtain quasi-uniform irradiation of the entire test detector. The reference detector consisted of a 2 × 2 × 3 mm^3^ LYSO crystal (EBO Crystal Inc., Shanghai, China) read using a 3 × 3 mm^2^ SiPM (Hamamatsu S14160-3050HS).

The experimental setup for the DOI resolution measurement is shown in Fig. 4b. The DOI resolution was measured using a reference detector consisting of a LYSO slab with dimensions of 40 × 20 × 1 mm^3^ and a single-channel photomultiplier tube (Hamamatsu R9800). The distance from the front of the slab detector to the source was 50 mm, whereas the distance between the side of the test detector and source was 40 mm. The irradiation beam width on the test detector was estimated to be approximately 1 mm, based on the measurement geometry. The slab detector and ^22^Na source were mounted on a moving stage so that different depths of the test LYSO arrays could be selectively irradiated by using coincidence between the slab detector and the test detector. Five depths—that is, 2, 6, 10, 14, and 18 mm from one end of the detectors—were irradiated for detectors 1 and 3. Seven depths—that is, 3, 7, 11, 15, 19, 23, and 27 mm from one end of the detectors—were irradiated for detectors 2 and 4.

All the measurements were performed in a light-tight box at an ambient temperature of 18 °C. The timing resolutions of the four dual-ended readout detectors and one single-ended readout detector using LYSO array 1 were measured at different SiPM bias voltages to determine the optimum bias voltage. The flood histogram, energy resolution, and DOI resolution were measured using only the optimum SiPM bias voltage.

### Data analysis

The x- and y-coordinates of the flood histograms measured by the SiPM arrays can be calculated as follows:

1 $$x_{1} = \frac{{X_{11} }}{{X_{11} + X_{12} }},\quad y_{1} = \frac{{Y_{11} }}{{Y_{11} + Y_{12} }}$$

2 $$x_{2} = \frac{{X_{21} }}{{X_{21} + X_{22} }},\quad y_{2} = \frac{{Y_{21} }}{{Y_{21} + Y_{22} }}$$

3 $$x = x_{1} + x_{2} , \quad y = y_{1} + y_{2}$$

where *X*_11_, *X*_12_, *Y*_11_, and *Y*_12_ are the four position-encoding energy signals of the first SiPM array placed in front of the detector, and *X*_21_, *X*_22_, *Y*_21_, and *Y*_22_ are the four position-encoding energy signals of the second SiPM array.

The energy measured using the SiPM arrays can be calculated as follows:

4 $$E_{1} = X_{11} + X_{12} + Y_{11} + Y_{12}$$

5 $$E_{2} = X_{21} + X_{22} + Y_{21} + Y_{22}$$

6 $$E = E_{1} + E_{2}$$

The DOI of a dual-ended readout detector can be obtained from the ratio of the energies measured using the two SiPM arrays placed at each end of the LYSO array, as follows:

7 $$\mathrm{DOI ratio}= \frac{{E}_{2}}{{E}_{1}+{E}_{2}}$$

The timing difference between each SiPM array and the reference detector can be treated as the timing of the SiPM array—that is, the curves of the timings measured by SiPM arrays 1 and 2 on the DOI ratios are measured; the curves can be fitted using a third-order polynomial function to obtain parameters used to correct the timing measured by each SiPM array [62, 63]; the average of the timings of the two SiPM arrays can then be used as the timing of the detector, as follows:

8 $$T= \frac{{T}_{1}+ {T}_{2}}{2}$$

To analyze the data, the flood histogram of a detector was first calculated using all measured data. A crystal look-up table for the detector was created from the analysis of the measured flood histogram. Second, the data were reanalyzed using the crystal look-up table, and the energy spectra of all individual crystals were obtained. The photopeak amplitude and FWHM of the 511 keV photopeak of each energy spectrum was obtained using a Gaussian fit. The crystal energy resolution was calculated by dividing the FWHM by the photopeak amplitude: Finally, using the data from the flood histogram of each detector, the DOI ratio histograms of all crystals in each detector and the timing spectra of all crystals in each detector were obtained by applying an energy window of 400–600 keV. The FWHM DOI resolution and CTR were obtained using a Gaussian fit. The DOI resolution was converted to mm using the DOI ratios measured at two depths close to the two SiPM arrays by assuming a linear relationship between the DOI ratios and depths. The CTR of two identical test detectors could be calculated by subtracting the CTR of the reference detector from the measured CTR of the test and reference detectors and multiplying the result by the root square of 2, as follows:

9 $$\mathrm{CTR}= \sqrt{2}\times \sqrt{{CTR}_{\mathrm{measured}}^{2}-{\mathrm{CTR}}_{\mathrm{ref}}^{2}/2}$$

The CTR of the two identical reference detectors was measured to be 103 ps for photopeak events.

## Results

### Flood histogram

**Fig. 5:**
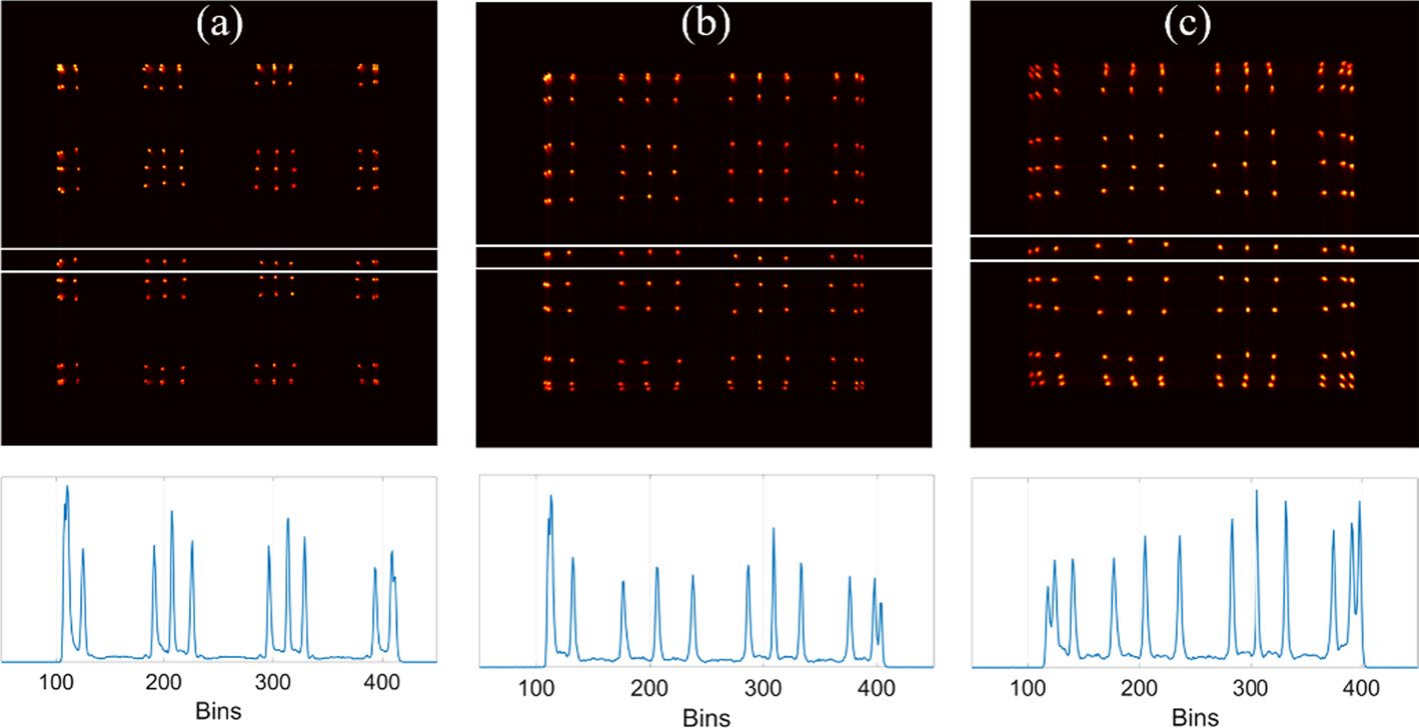
(Top) Flood histograms and (bottom) line profiles of the middle row of the crystals of detector 3 using 12 × 12 LYSO crystals of 1.95 × 1.95 × 20 mm^3^ measured **a** without a light guide, **b** with a 1-mm-thick glass light guide, and **c** with a 1-mm-thick light guide with grooves

The flood histograms and line profiles of the middle row of crystals of detector 3 measured without a light guide and with two different light guides are shown in Fig. 5. When the SiPM arrays are directly coupled to the 12 × 12 LYSO array with a crystal size of 1.95 × 1.95 × 20 mm^3^, the two rows/columns of the crystals at the edge are close to each other in the flood histogram and cannot be resolved. All other crystals can be clearly resolved, although they are not evenly distributed in the flood histogram. When 1-mm-thick glass plate light guides are placed between the crystal array and SiPM arrays, the flood histogram is more uniform, but the edge crystals cannot be resolved. When 1-mm thick glass light guides with grooves are placed between the crystal array and SiPM arrays, all crystals in the flood histogram can be resolved. Consequently, all measurements of detectors 1 and 2 were performed without a light guide, and all measurements of detectors 3 and 4 were performed with a light guide with grooves (unless otherwise stated).

**Fig. 6:**
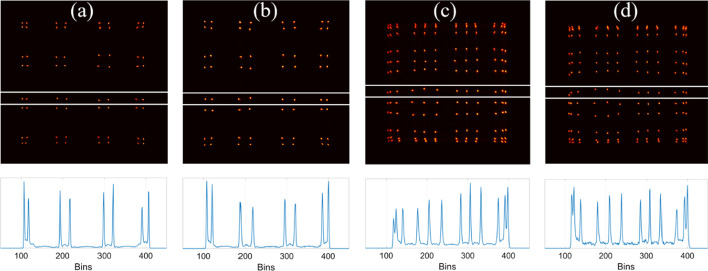
(top) Flood histograms and (bottom) line profiles of the middle row of crystals of the 4 detectors, **a** detector 1 using 8 × 8 LYSO array of 2.98 × 2.98 × 20 mm^3^ crystal size, **b** detector 2 using 8 × 8 LYSO array of 2.98 × 2.98 × 30 mm^3^ crystal size, **c** detector 3 using 12 × 12 LYSO array of 1.95 × 1.95 × 20 mm^3^ crystal size, and **d** detector 4 using 12 × 12 LYSO array of 1.95 × 1.95 × 30 mm^3^ crystal size. The flood histograms of detectors 1 and 2 are measured without light guides. The flood histograms of detectors 3 and 4 are measured using light guides with grooves

The flood histograms and line profiles of the middle row of crystals of the four detectors are shown in Fig. 6. All crystals of the four detectors can be clearly resolved. However, the crystal identification at the edges of the detectors is much better for the two detectors with a crystal size of 2.98 mm. The flood histograms of the two detectors with a crystal length of 30 mm are almost the same as those of the detectors with a crystal length of 20 mm.

### Energy resolution

**Fig. 7:**
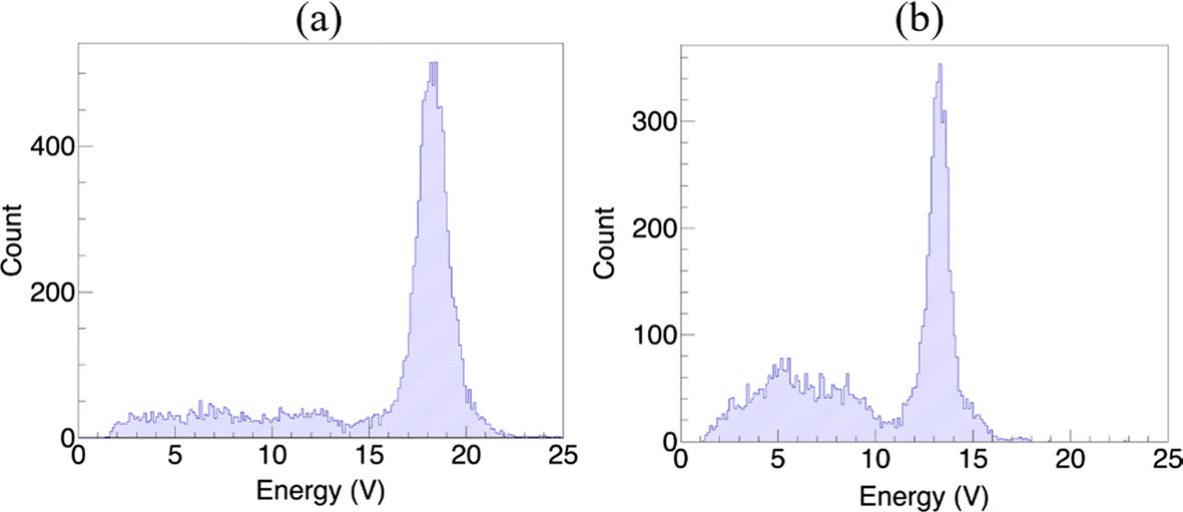
The energy spectra of (**a**), a middle crystal and **b** an edge crystal in the 3 × 3 × 20 mm^3^ crystal array

**Fig. 8:**
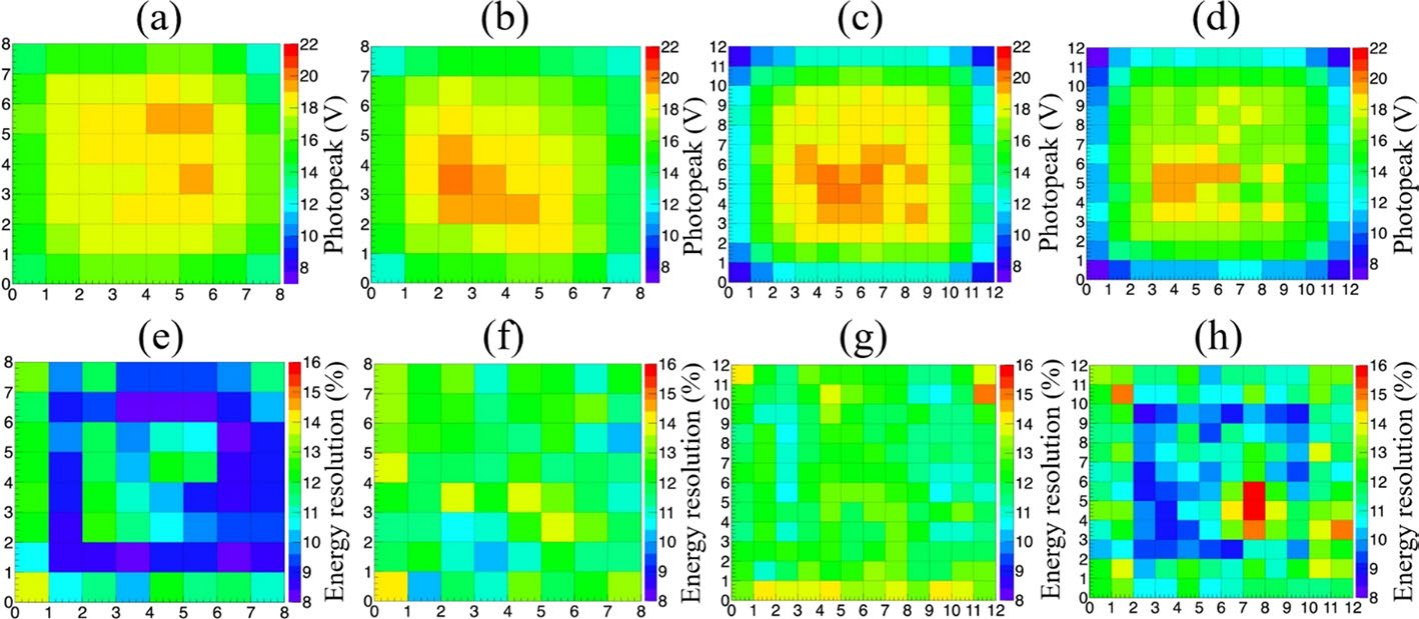
Photopeak amplitudes (top) of all individual crystals of the four detectors measured from the pulse height spectra of all depths, **a** detector 1, **b** detector 2, **c** detector 3, and **d** detector 4. Energy resolutions (bottom) of all individual crystals of the four detectors measured from the energy spectra of all depths, **e** detector 1, **f** detector 2, **g** detector 3, and **h** detector 4

The energy spectra of the single crystals in the 3 × 3 × 20 mm^3^ crystal array are shown in Fig. 7, while the photopeak amplitudes of all individual crystals of the four detectors are shown in Fig. 8. For all the detectors, the photopeak amplitudes of the middle crystals are slightly higher than those of the edge crystals. The mean photopeak amplitudes of the 3 × 3 × 20, 3 × 3 × 30, 2 × 2 × 20, and 2 × 2 × 30 mm^3^ LYSO crystal arrays are 17.0, 16.6, 15.7, and 14.8 V, respectively. The standard deviations are 1.5, 1.9, 3.0, and 2.8 V, respectively. For the two detectors with a 2.98-mm crystal cross-section, the uniformity of the photopeak amplitude is better than that of the two detectors with a 1.95-mm crystal cross-section, due to the use of the light guide with grooves filled with BaSO_4_. The energy resolutions of all individual crystals in the four detectors are shown in Fig. 8. The average crystal energy resolutions of the four detectors are 10.2 ± 0.2, 12.1 ± 0.3, 11.4 ± 0.3 and 11.7 ± 0.3% FWHM, respectively. The energy resolution only slightly degrades as the crystal size decreases and the crystal length increases.

### DOI resolution

**Fig. 9:**
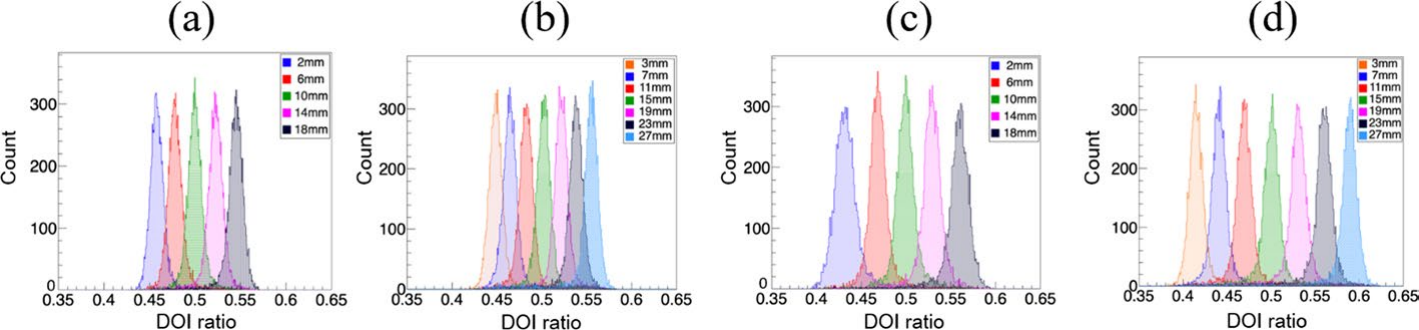
DOI ratio histograms of a middle crystal in the four detectors measured at different depths of 4-mm stepping size, **a** detector 1, **b** detector 2, **c** detector 3, and **d** detector 4. The depths measured for detectors 1 and 3 are 2, 6, 10, 14, and 18 mm from one end. The depths measured for detectors 2 and 4 are 3, 7, 11, 15, 19, 13, and 27 mm from one end

**Fig. 10:**
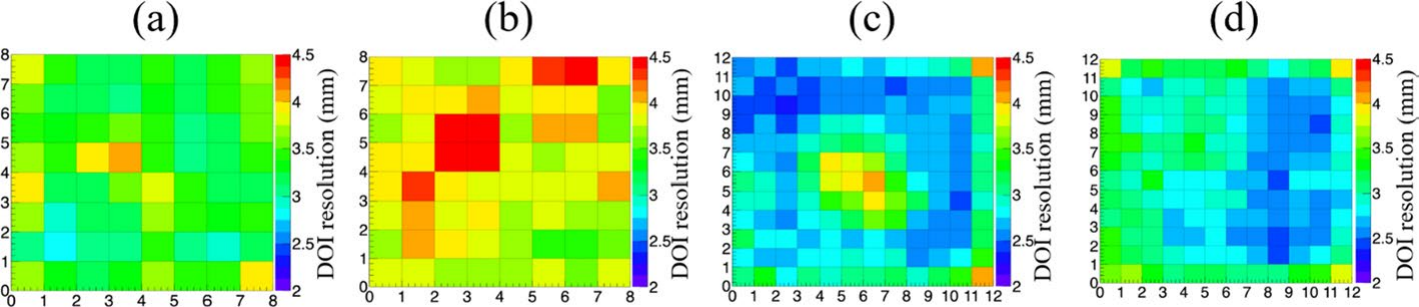
DOI resolutions of all individual crystals in **a** detector 1, **b** detector 2, **c** detector 3, and **d** detector 4

The DOI ratio histograms of the middle crystals of the four detectors, measured at different depths, are shown in Fig. 9. Compared to the detectors with a 2.98-mm crystal size, the ranges of the DOI ratio distribution of the detectors with 1.95-mm crystal size are wider, the DOI ratio increasing as the crystal length increases from 20 to 30 mm. The DOI ratio (centroid of the distribution) changes linearly with depth for all four detectors. The DOI resolutions of the individual crystals of the four detectors are shown in Fig. 10. The average DOI resolutions of the four detectors are 3.5 ± 0.2, 3.9 ± 0.3, 2.7 ± 0.3, and 3.0 ± 0.2 mm FWHM, respectively, as shown in Table 2, the DOI resolution degrading as the crystal size and length increase. It should be noted that the irradiation beam width—estimated to be ~ 1 mm from the experimental geometry—was not subtracted from the DOI resolution results in this work.

**Table 2:** Energy resolution, DOI resolution and CTRs of the four detectors

	Detector 1	Detector 2	Detector 3	Detector 4
Energy resolution (%)	10.2 ± 0.3	12.1 ± 0.3	11.4 ± 0.3	11.7 ± 0.3
DOI resolution (mm)	3.5 ± 0.3	3.9 ± 0.3	2.7 ± 0.3	3.0 ± 0.3
CTR/SiPM 1 (ps)	363 ± 6	475 ± 8	467 ± 8	543 ± 10
CTR/SiPM 2 (ps)	273 ± 5	339 ± 5	404 ± 7	465 ± 8
CTR/both, without correction (ps)	191 ± 3	240 ± 3	255 ± 4	285 ± 4
CTR/both, with correction (ps)	180 ± 2	214 ± 3	239 ± 3	263 ± 4

### Timing resolution

**Fig. 11:**
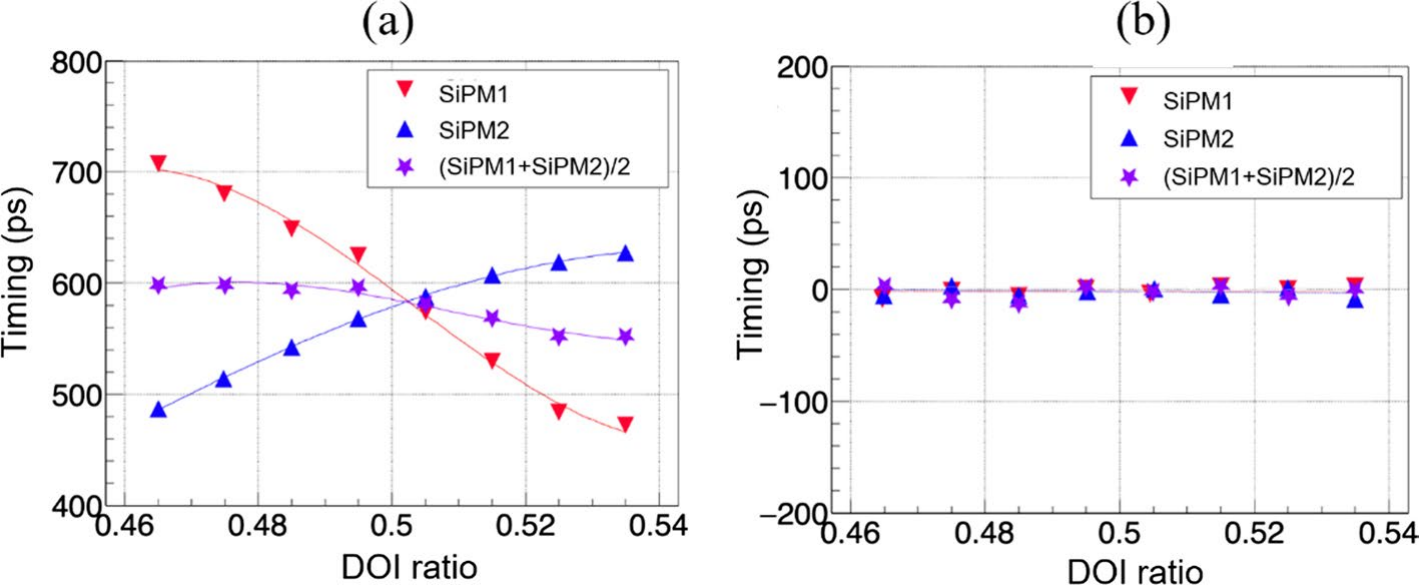
The timing (difference between the timing of a reference detector and the timing of the measured detector) vs. DOI ratio measured by SiPM 1, SiPM 2 and the average of the two SiPMs for detector 1, **a** before and **b** after depth dependence of timing correction

The timings measured by SiPM 1 and SiPM 2, and the average timing of the two SiPMs for different DOI ratios of detector 1—using an 8 × 8 LYSO array with a crystal size of 2.98 × 2.98 × 20 mm^3^—are shown in Fig. 11a. The timing measured by each SiPM changes with the DOI ratio because the travel time of the 511 keV photons (from entering the crystal array to the interaction point) and the travel time of the scintillation photons (from the interaction point to both SiPM arrays) change with depth. As the DOI ratio increases, the timing value measured by SiPM 1 changes more than that measured by SiPM 2, because as the depth increases (away from SiPM 1), the travel times of both the photons and scintillation photons from the interaction side to SiPM 1 increase, and the travel time of scintillation photons from the interaction site to SiPM 2 decreases. The curves of the timings measured using SiPM 1 and SiPM 2 for the DOI ratio can be fitted using a third-order polynomial function to obtain the correction parameters—consequently, the timing values for each event measured using SiPM 1 and SiPM 2 can be corrected to the timing values of depth 0 using the measured DOI ratio and the above correction parameters. The depth dependence of the timing values can be eliminated, and the timing resolution of the detectors can be improved. As shown in Fig. 11b, the depth dependence of the timings measured by each SiPM and both SiPMs is completely removed after calibration. Here, all CTR results are obtained with the correction of the depth dependence of timing pixel-by-pixel, unless noted otherwise.

**Fig. 12:**
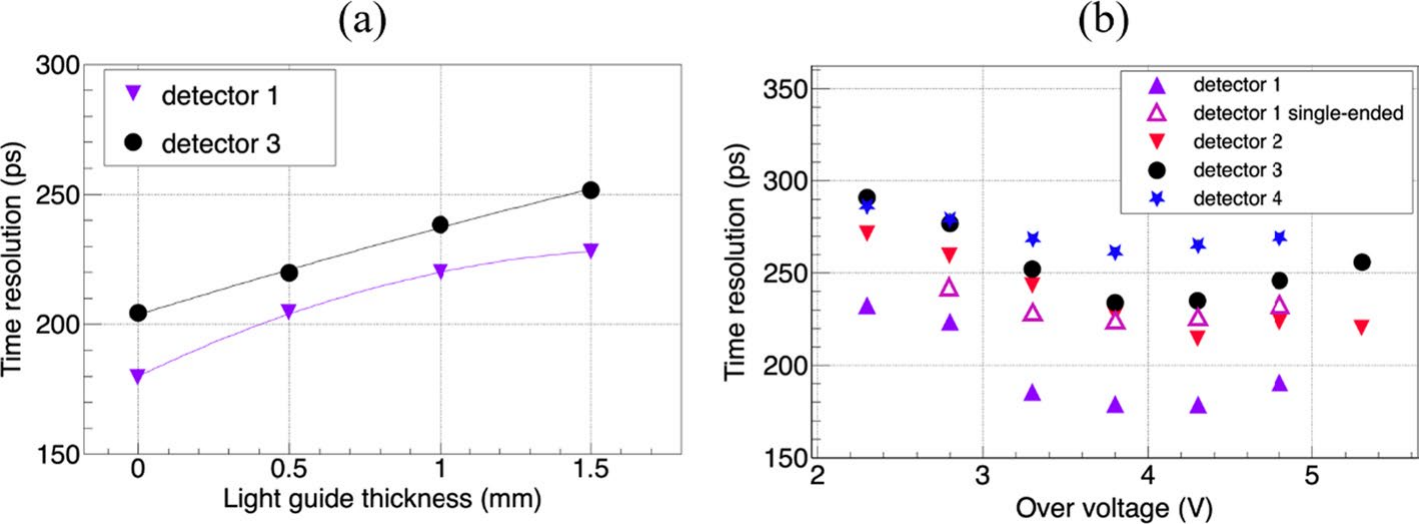
**a** CTRs of the detectors 1 and 3 as a function of light guide thickness, **b** CTRs of the five detectors as a function of the SiPM over voltage

Figure 12a shows the CTRs of detectors 1 and 3 as a function of the light-guide thickness. The timing resolution degrades as the thickness of the light guide increases. Both detectors exhibit the best timing resolution without the use of light guides. The CTRs of all four dual-ended readout detectors and one single-ended readout detector as a function of the SiPM overvoltage are shown in Fig. 12b. The breakdown voltage of the SiPMs can be obtained from the manufacturer’s datasheet. The CTR improves as the overvoltage is increased at lower voltages until an overvoltage of 4 V; above 4 V, the CTR degrades. The CTR of the dual-ended readout detector is approximately 20% better than that of the single-ended readout detector using the same LYSO array.

**Fig. 13:**
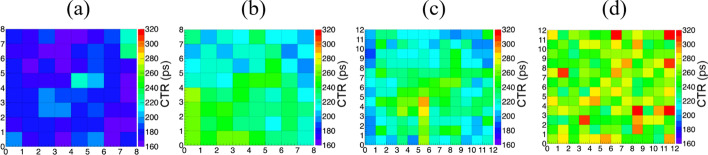
CTRs of all individual crystals of the four detectors, **a** detector 1, **b** detector 2, **c** detector 3, **d** detector 4

The CTRs of all individual crystals in the four detectors are shown in Fig. 13. The average CTRs of the four detectors measured using SiPM arrays 1 and 2, as well as both SiPM arrays with and without the depth dependence of timing correction, are shown in Table 2. The CTR degrades as the crystal size decreases and the crystal length increases. The CTR measured using SiPM 1 is worse than that measured using SiPM 2. For the dual-ended readout detector, the CTR measured using the timing information of both SiPMs is better than the CTR measured using each SiPM. The CTR with the depth dependence of the timing correction is better than that without correction. The best average CTRs of the four detectors obtained using both SiPMs after depth dependence of the timing correction are 180 ± 2, 214 ± 3, 239 ± 3, and 262 ± 4 ps.

## Discussion and conclusion

Detectors consisting of LYSO crystal arrays with ~ 2 and 3 mm crystal cross-sections, and 20 and 30 mm crystal lengths dual-ended read using SiPM arrays with a 6 × 6 mm^2^ pixel area were evaluated in this work. The timing and energy signals of the detectors were processed separately to obtain a good CTR, while simultaneously reducing the number of energy signals. Sixteen timing signals of a SiPM array were processed using a NINO ASIC, and CTRs of ~ 180–260 ps for the four detectors were obtained. The 16 energy signals of the SiPM array were converted to four position encoding energy signals using a commonly used row and column summing circuit. All crystals could be resolved from the flood histograms, and good energy and DOI resolutions were obtained.

The travel times of both 511 keV photons (from entering the scintillator array to the interaction point) and scintillation photons (from the interaction point to both SiPMs) change with depth. The change in the timing measured using SiPM 2 as the DOI ratio changed was smaller than that of SiPM 1—therefore, the CTR measured using SiPM 2 was better than that measured using SiPM 1. Moreover, because the sum of the travel distance from any interaction point to the two SiPM arrays was the crystal length, the depth dependence of the travel time of the scintillation photons could be removed by using the average timings measured using the two SiPMs placed at both ends of the scintillator array. Furthermore, the dependence of the timing values measured by each SiPM on the DOI ratios could be measured and corrected to improve the CTR of the dual-end readout detector.

The results of this study showed that the dual-ended readout detectors provided ~ 20% better CTR than the single-ended readout detectors using the same LYSO array and readout electronics. The detectors with larger crystal sizes provided better CTRs because the number of reflections of the scintillation photons on the crystal surfaces was reduced—that is, more scintillation photons could be detected. There was also no light guide used for the two detectors with large crystal cross-sections. For detectors with the same crystal cross-section, the CTR of the detector with a short crystal length was better, as expected, because the travel distance of the scintillation photons was shorter—that is, more scintillation photons could be detected. The correction of the depth dependence of the timings improved the CTRs of the 20 and 30 mm crystal length detectors by 5–6 and 8–11%, respectively. The timing of the detectors evaluated in this work was obtained using the NINO ASIC, which was originally designed for high-timing-resolution detectors of high-energy physics. The excellent timing properties of the NINO ASIC-based electronics played an important role in the PET detectors developed in this study achieving good CTRs.

In this study, DOI resolutions of 2.7–3.9 mm were obtained for the four detectors, which could be sufficient for building whole-body and total-body PET scanners. Polished crystal surfaces and BaSO_4_ reflectors were used based on our previous work on optimizing LYSO array parameters for high-resolution dual-ended readout PET detectors [31]. Moreover, because the crystal cross-section of this work was larger than the 1 × 1 mm^2^ crystal cross-section of the previous work [31], the DOI ratio ranges of the four detectors were smaller as the number of reflections of scintillation photons on the crystal surfaces decreases as the crystal cross-section increases, a small DOI ratio range leading to degradation of the DOI resolution. The DOI resolution of the detectors could be further improved by increasing the roughness of the crystal surfaces [64], which could lead to some degradation of the timing resolution. This should be further investigated in future work.

In this work, the crystal cross-section of the LYSO array with small crystals was only approximately one-third of the SiPM pixel size, nine crystals being coupled to the same SiPM pixel. Moreover, light guides were used to increase the scintillation photon spread and resolve edge crystals. It was established that the CTR decreased as the light-guide thickness increased, a possible reason being that a thicker light guide introduced more scintillation photon spread, reducing the maximum signal amplitude that could be measured by a single SiPM pixel. Consequently, the signal-to-noise ratio of the SiPM pixel degraded, as did the CTR of the detector. The small crystals could be resolved without using a light guide by using a SiPM array with a small pixel size to improve the CTR of the detector, increasing the number of SiPM pixels needed and the cost of the PET scanner.

Non-uniformity was also observed for the photopeak amplitude, energy resolution, timing value (data not shown), timing resolution, DOI ratio value (data not shown), and DOI resolution of the individual crystals in each detector. This was due to the non-uniformities of both the SiPMs and crystals, as well as the difference in the relative locations of the crystals within the SiPMs. The SiPMs in the array had slightly different breakdown voltages, the crystals in a crystal array also having a different light output owing to their non-uniform surface treatment during production. Moreover, the crystals located in the gaps between the SiPMs could exhibit poor performance, the edge crystals exhibiting different performance compared those in the middle owing to the penetration of the reflector by the scintillation photons and the leakage of the scintillation photons through the edge of the light guide. In current PET electronics, crystal-based energy, DOI, and timing calibrations can be performed [40, 65].

In the past, studies on dual-ended readout of segmented crystal arrays were focused primarily on the development of high-resolution depth-encoding small-animal PET detectors, only a few previous studies having been conducted on developing clinical PET detectors that simultaneously achieved high DOI and timing resolution [51–54]. In Refs. [51], [52], and [54], experiments were only performed on single crystals to optimize the crystal surface finishing to simultaneously obtain a good DOI and timing resolution. In Ref. [53], experiments were performed on a small 6 × 6 LYSO array with a crystal size of 2 × 2 × 20 mm^3^. A DOI resolution of 3.5 mm and timing resolution of 350 ps were obtained. In this work, the quantitative DOI and timing-resolution results of detectors with crystal arrays of different lengths (20 vs. 30 mm) and sizes (2 vs. 3 mm) were examined, a DOI resolution as good as 2.6 mm and a timing resolution as good as 180 ps being achieved. consequently, The crystal array dimensions of 24.6 × 24.6 × 20 mm^3^ and 24.6 × 24.6 × 20 mm^3^ could be suitable for use in a clinical PET scanner to achieve a balance between cost and counting rate performance.

Compared to traditional single-ended PET detectors, the dual-ended readout detectors developed in this work provided 2.7–3.9 mm DOI resolution and 20% better timing resolution. Although the DOI uncertainty effect was smaller for whole-body PET scanners than for small-animal PET scanners with smaller ring diameters and higher spatial resolutions, it becomes more serious as the crystal cross-section decreases and the axial field-of-view of the scanner increases, which is the current trend in whole-body PET scanner development. Consequently, the detector developed in this study could be a good candidate for use in the future to develop whole-body and total-body PET scanners capable of achieving uniform high spatial resolution, high sensitivity, and high timing resolution simultaneously.

One disadvantage of dual-ended readout detectors is that two photodetectors are required for one detector module, which increases the cost of both the photodetector and the electronics. In the current single-ended readout PET detector, the cost of the scintillator array is approximately three times that of the SiPM array, making the cost of the dual-ended readout PET detector acceptable, considering its benefits. Another disadvantage of dual-ended readout PET detectors is the presence of photodetectors and readout electronics in front of the scintillator array, which can cause some attenuation and scattering of the 511 keV photons and create gaps between detector modules in a PET scanner. Subsequently, a small-animal PET scanner using dual-ended readout detectors was developed by our group, a sensitivity of 16% at the center of the field of view, and a spatial resolution of less than 1 mm within the entire field-of-view was achieved for an energy window of 250–750 keV [40, 66]. Although it is more challenging, it was possible to use dual-ended readout detectors in a PET scanner. The gaps between the detector modules did not introduce visible artifacts into the images if a statistical image reconstruction algorithm was used, and the heat generated by the SiPM readout electronics was manageable.

## Data Availability

The datasets used and/or analyzed during the current study are available from the corresponding author on reasonable request.

## Abbreviations

TOF: Time-of-flight
PET: Positron emission tomography
SNR: Signal-to-noise ratio
DOI: Depth of interaction
SiPM: Silicon photomultiplier
FWHM: Full width at half maximum
CTR: Coincidence time resolution
BaSO_4_: Barium sulphate
ASIC: Application-specific integrated circuit
RMS: Root-mean-square
PCI: Peripheral component interconnect
